# Low mental health scores are associated with worse patient-reported outcomes and difficulty with return to work and sport after distal biceps repair

**DOI:** 10.1016/j.jseint.2020.12.015

**Published:** 2021-03-02

**Authors:** Thomas Yetter, Andrew G. Patton, Ahmed Mansi, Nicholas Maassen, Jeremy S. Somerson

**Affiliations:** a School of Medicine, The University of Texas Medical Branch, Galveston, TX, USA; b Department of Orthopaedic Surgery and Rehabilitation, The University of Texas Medical Branch, Galveston, TX, USA; c Department of Orthopaedic Surgery and Rehabilitation Medicine, University of Chicago, Chicago, IL, USA

**Keywords:** Distal, Biceps, Rupture, Repair, Mental health, Mental component score

## Abstract

**Background:**

Most patients have successful outcomes with minimal limitations after distal biceps repair, but a minority continues experiencing functional constraints. We hypothesize that low scores on a validated mental health measure correlate with worse patient-reported outcomes and increased difficulty with return to work and sport.

**Methods:**

We conducted a retrospective review of a consecutive series of patients who underwent distal biceps repair with a single-incision cortical button technique and immediate mobilization. Patient-reported outcome data were available at 1 year or later for 33 (85%) patients. The primary outcomes were American Shoulder and Elbow Surgeons-Elbow (ASES-E) score, Single Assessment Numeric Evaluation score, Visual Analog Scale for pain, Disabilities of the Arm, Shoulder and Hand Score (QuickDASH), and Veterans RAND 12 (VR-12) quality-of-life assessment.

**Results:**

All patients were male, with a median age of 49 years (range, 28-65). None had reruptures, and 1 (3%) had superficial wound dehiscence that healed without further surgery. Eleven (33%) had postoperative neuropraxia, 6 of which resolved completely. At latest follow-up, the median Visual Analog Scale was 0 (range, 0-5; mean, 1), and median ASES-E functional score was 36 (range, 24-36; mean, 34). Median Single Assessment Numeric Evaluation score was 92 (range, 41-100). The median QuickDASH was 5 (range, 0-50; mean, 11). More than half of the patients with VR-12 mental component score (MCS) < 50 (5 of 9, 56%) reported difficulty with work activities, compared with 4% (1 of 24) of patients with an MCS ≥ 50 (*P* = .001). Most patients (8 of 9, 89%) with an MCS < 50 also reported difficulty with return to sporting activities, compared with only 8% (2 of 24) of patients with MCS ≥ 50 (*P* < .0001). Patients with an MCS < 50 (n = 9) had significantly worse ASES-E functional scores (median, 34; range, 27-36) and QuickDASH scores (median 23, range 0-43), compared with those with an MCS ≥ 50 (ASES-E: median, 36; range, 24-36; *P* = .033; QuickDASH: median, 2; range, 0-50; *P* = .026). Most patients (17 of 24, 71%) with MCS ≥ 50 had a perfect score of 36 on the ASES-E functional outcome score, compared with only 22% (2 of 9) among patients with MCS < 50.

**Conclusion:**

Patients who undergo distal biceps repair show excellent functional patient-reported outcomes at 1-year and later follow-up. Lower scores on the VR-12 MCS are associated with worse patient-reported outcome scores and difficulty with return to work and sporting activities.

Incidence of distal biceps tendon rupture has been reported at 1.2 per 100,000 persons per year and typically affects men in their 40s.^5^^,^^21^ Anabolic steroid use, statin medications, and the use of tobacco products are all risk factors for rupture.^21^ Nonoperative management results in supination and elbow flexion weakness; therefore, surgical repair is commonly used to restore these deficiencies. Anatomic fixation has been shown to result in good strength and functional outcome scores.^4^^,^^7^ Tendon repair techniques have evolved over the years, with bone anchors, transosseous screws, and cortical buttons demonstrating good biomechanical strength with all techniques. In biomechanical studies, cortical buttons demonstrated the highest load to failure, allowing for earlier postoperative rehabilitation.^15^^,^^16^^,^^20^

However, some patients continue to have difficulty returning to work and sporting activities after surgical repair. The relationship between mental health and patient-reported outcomes and satisfaction in orthopedic surgery is a growing area of study.^22^^,^^26^ It has been shown that preoperative mental health scores have a stronger association with shoulder pain and functions scores than the actual tear size in full-thickness rotator cuff tears.^27^ Poor mental health is also associated with worse functional outcomes across orthopedic spine, trauma, sports medicine, and arthroplasty surgery.^3^^,^^9^^,^^10^ In addition, mental health has been shown to be predictive of patient return to work at full duty after rotator cuff surgery.^11^ To our knowledge, there has been no study that has analyzed the association of mental health scores and functional outcome scores after distal biceps repair. We hypothesize that low scores on a validated mental health measure are associated with worse patient-reported outcome measures and increased difficulty with return to work and sport.

## Materials and methods

We conducted a retrospective review of a consecutive series of patients who underwent distal biceps tendon repair between April 2016 and June 2019. Of 39 patients who underwent distal biceps repairs during this time, 33 patients were available for follow-up at the 1-year time point. The procedures were performed by 1 of 2 surgeons with fellowship training in shoulder and elbow surgery. One-year follow-up was selected for this traumatic condition based on published outcomes and reported timing of complications and reruptures within the first 4 months after surgery.^12^ Demographic data including age, gender, and follow-up time were collected. The primary outcomes in this study were the American Shoulder and Elbow Surgeons–Elbow (ASES-E) score, Single Assessment Numeric Evaluation (SANE) score, Visual Analog Scale (VAS) for pain, Disabilities of the Arm, Shoulder and Hand Score (QuickDASH), and Veterans RAND 12 (VR-12) quality-of-life assessment.

Diagnosis of distal biceps rupture is made by history and physical examination. The hook test is used to aid in diagnosis, and magnetic resonance imaging is used if the diagnosis is in question.^17^ Indications for surgery were ruptures of the distal biceps tendon in patients wishing to maximize function and strength.

### Surgical technique

With the elbow extended and in supination to protect the posterior interosseous nerve, a volar incision is made just ulnar to the brachioradialis. Full-thickness skin flaps are developed, and blunt dissection is performed through subcutaneous tissue. The brachioradialis is gently retracted laterally to protect the radial nerve motor branch. The lateral antebrachial cutaneous nerve is identified and retracted. Recurrent veins are retracted proximally, or isolated and coagulated with bipolar electrocautery. The interval is developed to the level of the joint capsule, with the forearm in supination. Using blunt dissection and bipolar electrocautery, the radial tuberosity is exposed. The biceps tendon is palpated and, with blunt dissection, delivered into the surgical field. If unable to identify or mobilize the proximal tendon, a second incision is made over the anteromedial distal arm. The ends are débrided and whip-stitched in grasping fashion with a looped, nonabsorbable #2 suture. The tendon is provisionally reduced to the level of the radial tuberosity and sized to ensure that it will pass into the drilled tunnel.

A guide pin is then placed slightly distal to the exact center of the insertion point. This is checked with fluoroscopy to ensure correct placement. The near cortex is reamed with a reamer matching the size of the measured tendon. The cortical button is attached to the end of the suture and the elbow flexed. The button is introduced through the bone, turned, and then flipped with appropriate tensioning to reduce the tendon to the bone. An interference screw is not used. The wound is then closed in layers and placed in a soft dressing with a compressive wrap.

### Postoperative rehabilitation protocol

After surgery, the patients are placed in a sling for 6 weeks with a soft dressing and instructions to come out of the sling for gentle passive range of motion as tolerated. Passive supination and active pronation is encouraged. For weeks 2 through 6, dressings are removed and patients begin regaining full extension with 2-finger 6-pack isometrics (excluding flexion). At 6 weeks, patients may progress to active range of motion as tolerated in pain-free range and begin 1-pound hammer curls. For weeks 12 through 16, patients may begin partial bodyweight pull-ups. After 4 months, patients continue to increase resistance with avoidance of eccentric loading until after 6 months. They are kept out of contact sports for 6 months.

### Statistical analysis

Descriptive statistics were reported with median, mean, and range. Intergroup comparisons were performed using a nonparametric Wilcoxon/Kruskal-Wallis rank sum test. Statistical significance was defined as *P* < .05 for all statistical analyses.

## Results

The 33 patients were followed up for a mean of 2 years (range, 1-4). All patients were male, with a median age of 49 years (range, 28-65). Median time from injury to surgery was 18 days (range, 3-380; mean, 49). Twenty patients had surgery in the acute phase (< 21 days after injury), 8 patients had subacute repair (between 21 and 90 days after injury), and 5 patients had chronic repair (> 90 days after injury).

Patients were stratified into those with a low VR-12 mental component score (MCS) of < 50 (n = 9) and those with a high VR-12 MCS of > 50 (n = 24; Table I). The groups did not significantly differ in age, time from injury to surgery, or postoperative neuropraxia rates.

**Table I:** Patient-reported outcomes at latest follow-up.

Parameter	All subjects (N = 33)	Mental component score < 50 (n = 9)	Mental component score ≥ 50 (n = 24)
Median, range (mean)			
Visual Analog Scale	0, 0-5.2 (1.1)	1, 0-5.2 (1.6)	0, 0-5 (.9)
ASES-E function score	36, 24-36 (34)	34, 27-36	36, 24-36∗
QuickDASH	5, 0-50 (11)	23, 0-43	2, 0-50∗
Single Assessment Numeric Evaluation	92, 41-100 (87)	86, 51-100 (83)	96, 41-100 (88)
Number of subjects (%)			
Perfect ASES-E function score	19 (58%)	2 (22%)	17 (71%)∗
Difficulty with work	6 (18%)	5 (56%)	1 (4%)∗∗∗
Difficulty with sport	10 (30%)	8 (89%)	2 (8%)∗∗∗

*ASES-E*, American Shoulder and Elbow Surgeons–Elbow; *DASH*, Disabilities of the Arm, Shoulder, and Hand.

∗*P* ≤ .05, ∗∗*P* ≤ .01, ∗∗∗*P* ≤ .001.

### Complications and reoperations

No patients had reruptures. One patient (3%) had superficial wound dehiscence that healed without further surgery. Eleven patients (33%) had postoperative subjective neuropraxia; at latest follow-up, 6 patients had complete resolution, whereas the remaining 5 (15%) had persistent sensory neuropraxia. No patients had reoperations at the repair site. One patient with an acute rupture had excellent results at his repair site but developed worsening postoperative pain at his proximal biceps. His symptoms responded completely to an ultrasound-guided steroid injection at the proximal biceps sheath, but then recurred. He underwent subsequent shoulder arthroscopy with proximal subpectoral biceps tenodesis 4 months after his distal biceps repair, resulting in relief of pain.

### Outcome measures

At latest follow-up, the median VAS was 0 (range, 0-5.2; mean, 1.1), and the median ASES-E functional score was 36 (range, 24-36; mean, 34). The median SANE score was 92 (range, 41-100; mean, 87). The median QuickDASH was 5 (range, 0-50; mean, 11). The median VR-12 MCS score at latest follow-up was 56 (range, 35 to 63; mean, 53).

**Figure 1:**
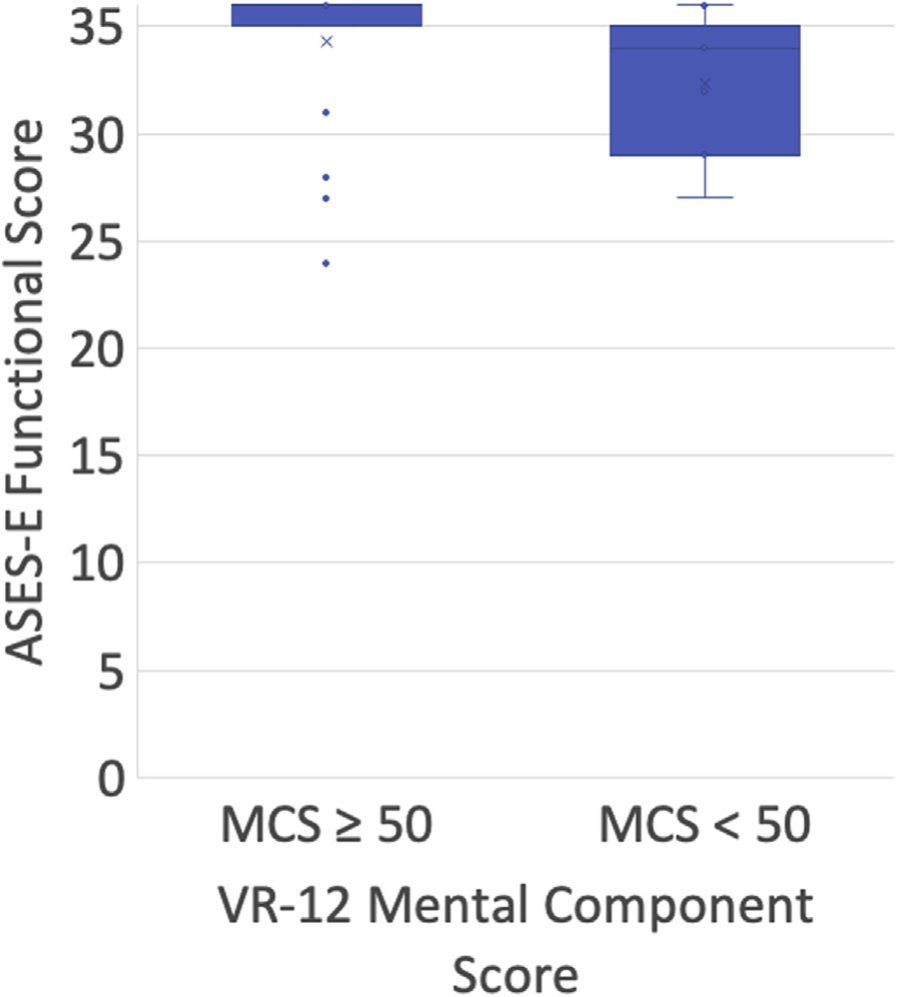
American Shoulder and Elbow Surgeons-Elbow (ASES-E) functional scores by Veterans RAND (VR-12) mental component score.

**Figure 2:**
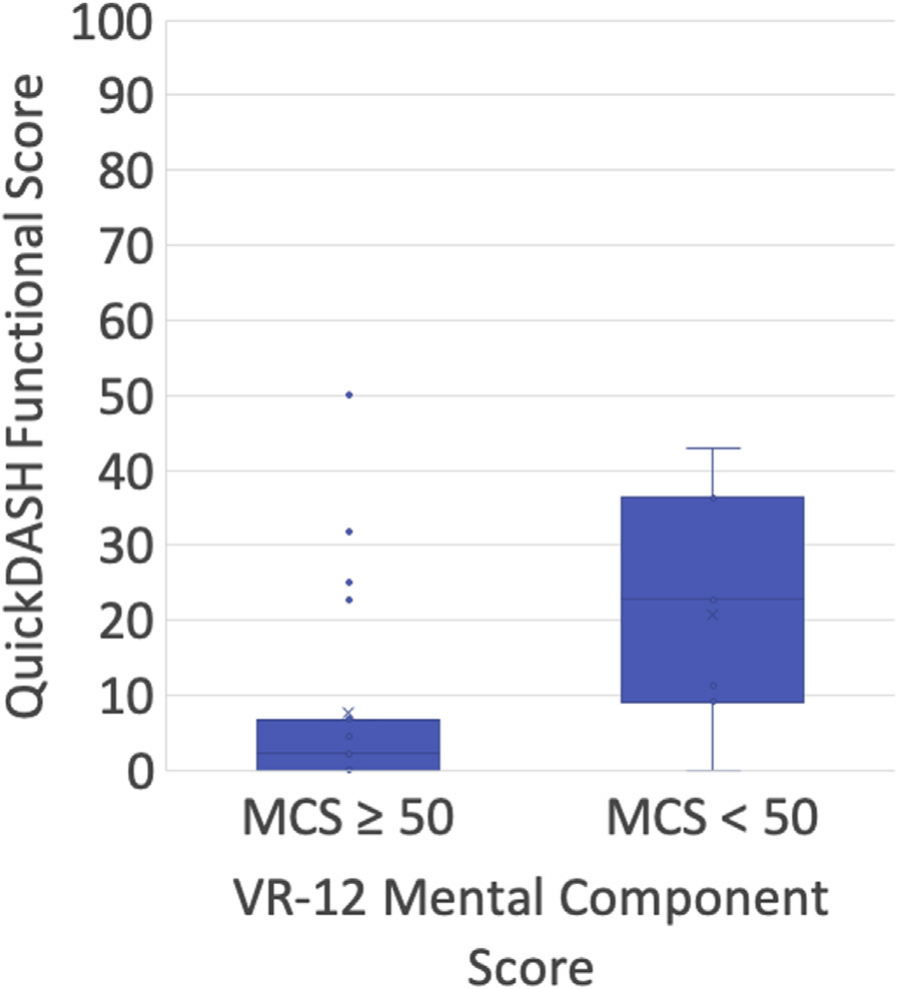
Disabilities of the Arm, Shoulder and Hand (QuickDASH) functional scores by Veterans RAND (VR-12) mental component score.

Fifty-six percent (5 of 9) of patients with VR-12 MCS < 50 at latest follow-up reported difficulty with work activities, compared with 4% (1 of 24) of patients with an MCS ≥ 50 (*P =* .001). Eighty-nine percent (8 of 9) of patients with an MCS < 50 also reported difficulty with return to sporting activities, compared with only 8% (2 of 24) of patients with MCS ≥ 50 (*P <* .0001). Patients with an MCS < 50 had significantly worse ASES-E functional scores (median, 34; range, 27-36; Fig. 1) and QuickDASH scores (median, 23; range 0-43; Fig. 2) compared with those with an MCS ≥ 50 (ASES-E: median, 36; range, 24-36; *P =* .033; QuickDASH: median, 2; range, 0-50; *P =* .026). Most patients (17 of 24, 71%) with MCS ≥ 50 had a perfect score of 36 on the ASES-E functional outcome score, compared with only 22% (2 of 9) among patients with MCS < 50 (*P =* .012). There was no significant difference between the VAS and SANE scores between the groups.

## Discussion

Patients who undergo distal biceps repair show good strength and functional outcome scores.^4^^,^^7^ Our patient group reported satisfactory functional outcomes at 1 year and later follow-up. In most patients, patient-reported outcome scores were excellent. Few patients had difficulty with work or sport. The 15% complication rate of persistent subjective neuropraxia in our cohort matched reported rates in the literature of 12% to 13%.^1^^,^^6^^,^^25^ This complication is more common in single-incision surgeries and is likely the result of traction during exposure of the radial tuberosity.^5^^,^^8^^,^^18^ No tendon reruptures or reoperations at the surgical site were observed in our cohort, in line with rates reported in previous case series of 0% to 5%.^2^^,^^13^^,^^14^^,^^23^

The primary limitations of this work are those inherent to a retrospective review. Although we present results from a consecutive series with over 80% follow-up at a minimum of 1 year, there remains the possibility of selection bias. In addition, we did not have preoperative VR-12 scores or functional outcome measures available for analysis. Further study is needed to determine whether preoperative indices of mental health are predictive with regard to functional outcomes and return to work or sport.

There have been relatively few published consecutive series of cortical button distal biceps repair that include patient-reported outcomes at minimum 1-year follow-up. Previous studies mainly focused on postoperative strength, range of motion, and complication rates for outcome analysis. Secondary outcome analysis in some series included functional outcome scores, such as the DASH and Mayo Elbow Performance Score; however, we are not aware of literature that reports associations between mental health and outcomes after distal biceps repair.^2^^,^^13^^,^^14^^,^^23^ While the body of evidence supporting the connection between mental health and postoperative outcomes is growing, literature assessing mental health interventions and surgical outcomes is still sparse. An ongoing randomized controlled trial is assessing the use of a patient telephone support program in total knee arthroplasty outcomes; however, results have not yet been published.^3^ The effectiveness of psychiatric counseling or social support programs in patients with low mental health scores for improving surgical outcomes remains unknown.

In this work, patients with VR-12 MCS scores < 50 had significantly worse reported QuickDASH and ASES-E functional scores, as well as a lower percentage of perfect ASES-E scores than patients with VR-12 MCS scores ≥ 50. The strongest associations were found in difficulty returning to work and sport. Although a causative relationship cannot be determined from this study, our results support the association between poor mental health scores and worse postoperative patient-reported outcomes in orthopedic surgeries reported in the literature.^10^^,^^19^^,^^24^ Interestingly, there was no difference between 1-year VAS or SANE scores between the groups. This likely eliminates pain and perceived limb disfunction as contributory factors for the difference in the reported outcomes and difficulty in return to work and sport. Our results support the growing body of evidence that mental health is strongly associated with the success of orthopedic surgical outcomes and patient satisfaction.

## Conclusion

Lower scores on the VR-12 MCS are associated with worse patient-reported outcomes scores and increased reported difficulty with return to work and sporting activities at 1 year after distal biceps repair. More study is needed to determine whether perioperative mental health interventions could play a role in improving outcomes. However, functional results after distal biceps repair were generally excellent, and complications were relatively minor in nature.

## Disclaimers:

*Funding:* No funding was disclosed by the authors.

*Conflicts of interest:* Jeremy S. Somerson reports educational support from 10.13039/100007307Arthrex, Medinc of Texas, and DJO/Encore, outside the scope of this project. The other authors and their immediate family, and any research foundation with which they are affiliated, did not receive any financial payments or other benefits from any commercial entity related to the subject of this article.
